# An Online Continuous Human Action Recognition Algorithm Based on the Kinect Sensor

**DOI:** 10.3390/s16020161

**Published:** 2016-01-28

**Authors:** Guangming Zhu, Liang Zhang, Peiyi Shen, Juan Song

**Affiliations:** School of Software, Xidian University, Xi’an 710071, China; gmzhu@xidian.edu.cn (G.Z.); liangzhang@xidian.edu.cn (L.Z.); songjuan@mail.xidian.edu.cn (J.S.)

**Keywords:** continuous human action recognition, online segmentation, maximum entropy Markov model, Kinect

## Abstract

Continuous human action recognition (CHAR) is more practical in human-robot interactions. In this paper, an online CHAR algorithm is proposed based on skeletal data extracted from RGB-D images captured by Kinect sensors. Each human action is modeled by a sequence of key poses and atomic motions in a particular order. In order to extract key poses and atomic motions, feature sequences are divided into pose feature segments and motion feature segments, by use of the online segmentation method based on potential differences of features. Likelihood probabilities that each feature segment can be labeled as the extracted key poses or atomic motions, are computed in the online model matching process. An online classification method with variable-length maximal entropy Markov model (MEMM) is performed based on the likelihood probabilities, for recognizing continuous human actions. The variable-length MEMM method ensures the effectiveness and efficiency of the proposed CHAR method. Compared with the published CHAR methods, the proposed algorithm does not need to detect the start and end points of each human action in advance. The experimental results on public datasets show that the proposed algorithm is effective and highly-efficient for recognizing continuous human actions.

## 1. Introduction

Human action recognition is a crucial and challenging task in many research and application fields, such as video surveillance [1,2], human-robot interactions [3]. With the aging of the population, service robots will play an important role in our daily life in the future. Observing and reacting to human actions automatically will become an essential skill for service robots [4]. Thanks to the advent of inexpensive depth sensors, such as the Microsoft Kinect sensor, huge progress has been made in daily activity recognition [5]. Human action recognition may become an ordinary application in our future life, partly owing to affordable Kinect sensors. 

Human skeletal data can be extracted from RGB-D data which are captured by a Kinect sensor [6], and human actions can be modeled as a continuous evolution of human skeletal joints [7]. However, most of the published human action recognition methods up to now mainly focus on segmented and unified action classification, *i.e.*, to identify the category of each data sequence which only contains one single human action performed by one single person [8,9,10,11]. Public datasets, such as MSRAction3D [12], MSRC-12 [13], and CAD-60 [14], also supply data sequences which have been segmented according to action categories and performers. Therefore, the state-of-the-art human action recognition algorithms may encounter unanticipated problems in practical applications. In practical applications, one RGB-D data sequence may contain several kinds of human actions which are not segmented in advance, so it may be necessary to detect the start and end frames of each human action, if we want to apply some published methods to the practical human daily life. Sliding window matching is a simple and effective method to recognize human actions from one continuous data sequence [13], but it is a tricky problem to decide the sliding window size. Besides, human actions are continuous and people may change their actions frequently, thus there may not be obvious boundaries between two different kinds of human actions [15]. Therefore, it is necessary to research on online continuous human action recognition (CHAR) algorithms for practical applications of service robots. 

**Figure 1:**
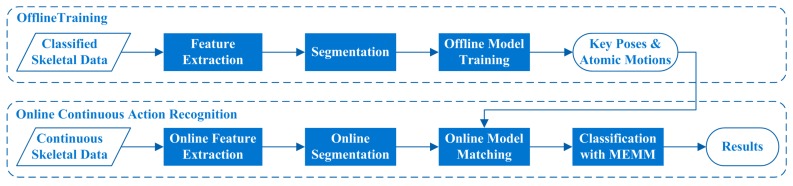
Overview of the proposed algorithm.

This study focuses on online CHAR algorithm which is aimed to recognize human actions from RGB-D data sequences which may include several kinds of human actions. As the further research of our previous work [16], we still represent each human action by a sequence of key poses and atomic motions in a particular order. We further introduce online segmentation method, online model matching method, and online classification method with Maximum Entropy Markov Model (MEMM) [17] in this study, to fulfill online continuous human action recognition. The overview of the proposed algorithm is displayed in Figure 1. The offline training processes are performed on classified skeletal data which are extracted from RGB-D images. The online continuous action recognition process can recognize each human action from continuous skeletal data when the skeletal data are being generated frame by frame. The feature sequences computed from skeletal data are segmented dynamically into pose feature segments and motion feature segments online, the online model matching method is performed on each segment, and a variable-length MEMM method is utilized to recognize human actions based on the matching results. The variable-length MEMM method not only can ensure the recognition effectiveness of similar human actions, but also can improve the recognition efficiency of discriminatory human actions. 

Compared with the CHAR methods [18,19] which need to detect the start and end points of human actions beforehand, the proposed algorithm does not detect the start and end points of human actions explicitly. The proposed algorithm divides feature sequences into pose feature segments and motion feature segments, and the potential-difference-based segmentation method ensures that almost all the boundaries of human actions can be conformed with boundaries of the first and last segments within each human action’s feature sequence. Thus, the correct classification of feature segments means correct continuous human action recognition. Different from the CHAR method [20] which detects action zones (*i.e.*, the most discriminative segments) before the recognition process, the proposed method does not extract the discrimination property of each feature segment beforehand. The utilized variable-length MEMM method can take full use of the discrimination or neutrality property of feature segments implicitly, and is more effective and efficient than the sliding-window-based CHAR method [13] which has fixed window size. The proposed algorithm is based on the online segmentation and online recognition of segments, it is very efficient and can be executed online and in real-time when skeletal data are being generated frame by frame.

The remainder of the paper is organized as follows: Section 2 reviews the related work about human action recognition briefly. In Section 3, the proposed algorithm is elaborated in detail. In Section 4, the experimental results and discussions are presented to demonstrate the advantages of the proposed algorithm. At last, Section 5 gives the conclusions and suggests future work.

## 2. Related Work

It may be trivial for humans to determine the relevant segments from one continuous RGB-D image sequence, but it is difficult for an automated human action recognition system. This is partly why numerous published human action recognition methods mainly focus on classification of segmented and unified data sequences which only contains one human action each. In this section, we divide published human action recognition methods into two categories: segmented human action recognition methods and continuous human action recognition methods. The former are aimed at identifying the category of segmented and unified data sequences, and the latter try to recognize human actions from continuous data sequences which contain multiple kinds of human activities. 

### 2.1. Segmented Human Action Recognition

A human action can be represented by a sequence of key poses [21], thus codebook-based human action recognition methods emerge [22,23]. In [24], key poses are clustered from pose feature vectors which are composed of positions of human joints relative to the torso joint. In [25,26], position offsets of 3D skeletal joints are computed and assembled using a bag-of-words framework for human action recognition. In order to keep the view invariance of pose feature representation, the skeletal quad which encodes the relative position of joint quadruples as local skeleton descriptors is proposed in [27], the proposed skeleton features are 6D view-invariant. In [28], hierarchical covariance matrices of skeletal joint locations are computed as discriminatory descriptors for each data sequence. In [29], static posture, motion property, and overall dynamics are combined as EigenJoints, and non-parametric Naive-Bayes-Nearest-Neighbor method is employed to classify multiple human actions. In [30,31], moving poses and elementary moving poses are proposed as efficient 3D kinematics descriptors for human action recognition, respectively. In addition to the above-mentioned feature representation methods which use relative joint locations or relative joint angles as skeletal features, histogram-based feature representation methods are also proposed, such as histogram of oriented displacements (HOD) [32], histogram of 3D joint locations (HOJ3D) [33], and histogram of oriented 4D normal (HON4D) [34].

Different from the abovementioned methods, we believe that one human action can be represented by a sequence of key poses and atomic motions in a particular order [16]. Key poses denote still poses and poses with tiny movements, and atomic motions indicate significant movements. In other words, key poses denote normal states of one human action, and atomic motions represent the transition process between each two key poses [16]. 

### 2.2. Continuous Human Action Recognition

In continuous human action recognition, data sequences are not segmented according to action categories in advance. Thus, boundaries between two kinds of human actions within one data sequence are unavailable. Sliding window matching is a simple and effective method to recognize human actions from one continuous data sequence [13]. In [20], the authors try to extract action zones which correspond to the most discriminatory segments, and employ a sliding and growing window approach for continuous human action recognition. In [35], a latent-dynamic conditional random field is utilized with a temporal sliding window to perform continuous gesture recognition. In [36], a sliding window is employed to build frame-wise Fisher vectors which will be classified by a multi-class SVM. However, it is a tricky problem to decide the sliding window size for such methods. A generative model based on the bag-of-words representation and the translation and scale invariant probabilistic Latent Semantic Analysis model (TSI-pLSA) is proposed in [18], the start and end frames of one human action are detected according the posterior probability using a threshold-based method. In [19], the authors use Hidden Markov Model (HMM) based action modeling method to model various human actions, and employ action spotter method to filter meaningless human actions and to detect the start and end points of human actions. In such methods, each human action can be recognized after the start and end points of the action are detected. Besides, methods which do not detect the start and end points of each human action beforehand emerge. In [37], discriminative orderlets are proposed to encode spatial configuration of a group of skeleton joints, and local occupancy pattern (LOP) orderlets are defined to encode object features. These frame-level orderlets are used for online continuous recognition of human-object interactions. In [38], a visual alignment technique named dynamic frame warping is proposed, which performs isolated recognition based on aligning a test sequence with a model sequence. In [39], a graphical model is designed to systematically concatenate different separately trained cyclic hidden Markov models for continuous action recognition. In [40], a probabilistic graphical model with substructure transition model and discriminative boundary model is proposed for continuous action recognition.

In this study, we do not detect the start and end points of each human action explicitly. We segment feature sequences online, and employ a variable-length MEMM method to recognize human actions based on the online model matching results of feature segments. 

## 3. Proposed Algorithm

In this section, the proposed algorithm will be described in detail. Firstly, the feature extraction method which has been proposed in our previous work [16] is elaborated again to keep the completeness of this academic paper. Secondly, the online segmentation method based on potential differences of feature sequences is described. Thirdly, the offline model training and the online model matching processes are presented. Lastly, the classification method based on the variable-length MEMM is stated in detail.

### 3.1. Feature Extraction

Since human body can be modeled as an articulated system of rigid segments [7], skeletal data extracted from RGB-D images can be utilized to represent some human actions. Thus, only skeletal data is used to extract features in this study. However, skeletal data may be unstable and noisy, and even “Corrupted” skeletal data may emerge due to occlusion of human body [41]. Therefore, a moving average filter is utilized to smooth skeletal data in advance.

**Figure 2:**
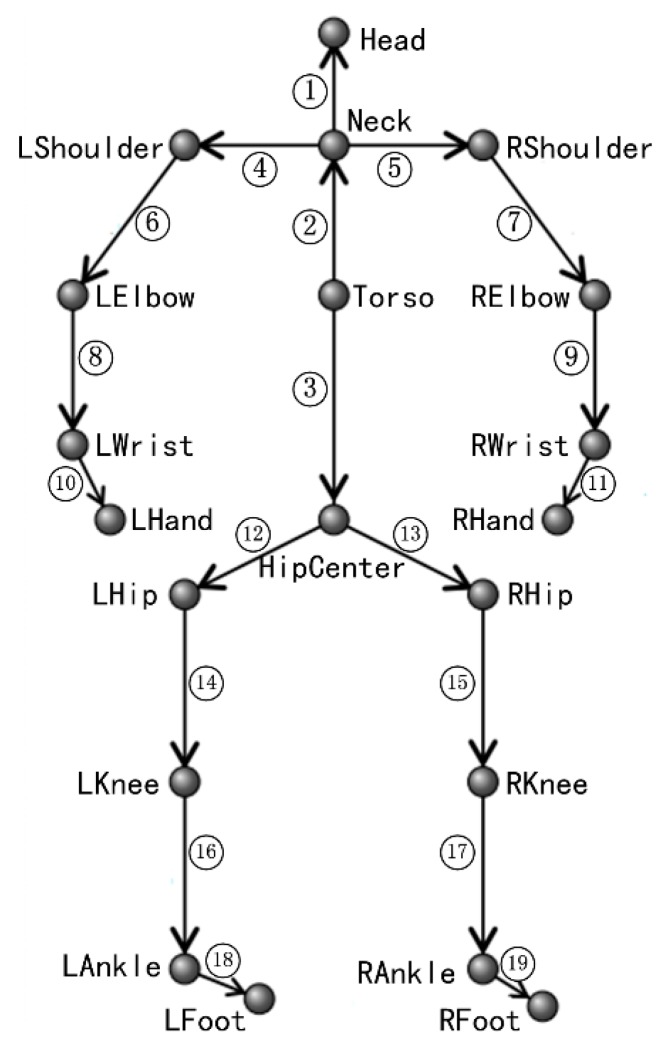
The NRO features based on human skeleton.

In order to take full use of dynamics models of human joints, Normalized Relative Orient (NRO) [16] for each human joint is computed as features. The NRO of one human joint is computed relative to the joint that it rotates around. For example, as displayed in Figure 2, number ⑥ indicates left elbow joint’s NRO which is computed relative to left shoulder joint. Let $L_{i}=(l_{x}^{i},l_{y}^{i},l_{z}^{i})$ denote the location of joint *i* in the 3D world coordinate system, the NRO of joint *i* relative to joint *j* can be computed as:

(1) $$F_{NRO}=\frac{L_{i}-L_{j}}{\left\|L_{i}-L_{j}\right\|}$$

where ‖⋅‖ denotes the Euclidean distance. It can be concluded from the Equation (1) that NRO is insensitive to human subject’s height, limb length, and distance to the camera.

The NROs of all human joints can be computed as illustrated in Figure 2. The NRO of the joint at the head of one arrow is computed relative to the joint at the tail of the arrow. It is necessary to point out that the skeletal data provided by the PrimeSense Natural Interaction Middleware (NiTE) have only 15 joints (without 5 joints, *i.e.*, HipCenter, LWrist, RWrist, LAnkle, and RAnkle) [14], thus the NROs labeled with ③, ⑩, ⑪, ⑱, and ⑲ will be removed when dealing with NiTE skeletal data. 

The NROs take full use of dynamics models of human joints. More specifically, the dynamics models of human joints in the world coordinate system are complex, even the dynamics models in the local coordinate system whose origin of coordinates is Torso [14] or HipCenter [8] are complex too, since movements of human joints are not independent from each other. But, the dynamics models represented by NROs are only simple rotation motion. Besides, such feature representation method is more conductive to human action imitation researches. This is partly why we choose NROs as features in our current and future researches, different from the published methods. 

### 3.2. Online Segmentation

Intuitively, a human action can be modeled by a sequence of key poses and atomic motions in a particular order [16]. In order to extract key poses and atomic motions, it is necessary to divide feature sequences into pose feature segments and motion feature segments first. A part of the published methods [22,23,24,25,26,27,28] do not distinguish key poses and atomic motions. They may take both static information and motion information into consideration simultaneously, but they combine them into one feature vector, and temporal sequence information among static poses and motion processes is lost more or less. In this study, an online segmentation method based on potential differences of feature sequences is proposed.

Given a feature sequence *S* = (*F*_1_,*F*_2_,…,*F_i_*,…) where each feature vector *F_i_* is composed of the NROs indicated by numbers with ring in Figure 2. Define the potential energy of the *i*^th^ feature vector as:

(2) $$E_{p}(i)={\left\|F_{i}-F_{r}\right\|}^{2}$$

where *F_r_* is the referred feature vector which can be the NROs of the pose “stand at attention” or other normal poses. Then, the potential difference of the *i*^th^ feature vector can be computed as:

(3) $$E_{d}(i)=E_{p}(i)-E_{p}(i-1)$$

Furthermore, define the sign of *E_d_*(*i*) as:

(4) $$S_{i}=\{\begin{array}{r} 1,\\ 0,\\ -1, \end{array}\begin{array}{l} E_{d}(i)\geq E_{\min}\\ \left|E_{d}(i)\right|<E_{\min}\\ E_{d}(i)\leq -E_{\min} \end{array}$$

where *E_min_* is an empirical parameter larger than zero. Then, segments wherein all potential differences of feature vectors have *S_i_* = 0, *i* = *i_seg_start_*,…,*i_seg_end_* are labeled as pose feature segments, and the others are motion feature segments.

**Figure 3:**
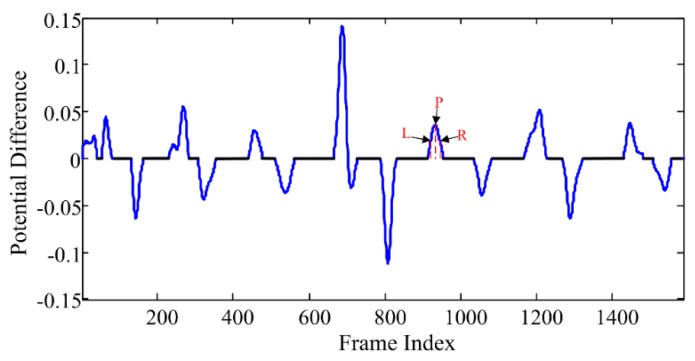
Segmentation result of the action “drinking water” in CAD-60 dataset. The black lines indicate the pose feature segments, and the blue curves indicate the motion feature segments. The L/P/R indicate the left hillside/the peak/the right hillside positions of one curve respectively, which will be used in the atomic motion extraction process in Section 3.3.1.

Given the above definitions, the online segmentation method can be summarized as in Algorithm 1. The segmentation results of the action “drinking water” in CAD-60 dataset using the proposed online segmentation method are displayed in Figure 3. It can be found from Figure 3 that pose feature segments and motion feature segments appear in turn.

**Algorithm 1.** The online segmentation process
**Initial**: Start one segment from the frame start, let $F_{start}$ and denote the feature vector of the frame and the sign of $E_{d}(start)$, respectively.
**For** each feature vector $F_{i}$ (The subscript i indicate the frame index) 
   Step 1: Compute the potential difference $E_{d}(i)$ and its sign $S_{i}$. 
   Step 2: 
   **If** $S_{i}==S_{start}$ 
     **If** $i-start>LP_{\max}\&S_{start}==0$ /*$LP_{\max}$ is the maximal length of pose feature segments. */ 
	 Complete the segment at the frame *i*–1; /* Pose feature segments. */ 
     **Else** 
	 Continue; 
     **End** 
   Else /* $S_{i}\neq S_{start}$ */ 
     **If** $i-start<LM_{\min}\&S_{start}\neq 0$**** /*$LM_{\min}$ is the minimal length of motion feature segments. */
	 $S_{start}=S_{j}$; /* Eliminate tiny motion feature segments. */ 
	 Continue; 
     **Else**
	 Complete the segment at the frame i−1; /* The segment type is decided by $S_{start}$. */ 
	 **If** $S_{i}*S_{start}==-1$ /* Two adjacent motion feature segments */ 
             Insert one pose feature segment which is only composed of the features $F_{i-1}$ and $F_{i}$; 
	 **End** 
     **End** 
  **End**
  Step 3: Start one new segment from the frame *i*, let $F_{start}=F_{j}$ and $S_{start}=S_{j}$;
**End**

### 3.3. Model Training and Matching

After the segmentation process, pose feature segments and motion feature segments are obtained. Let *s* = 1,2,… denote the sequence number of pose feature segments. According to the online segmentation method, one motion feature segment is located in the middle of two pose feature segments, as displayed in Figure 3, thus the *s*^th^ motion feature segment is located in the middle of the ${\left(s-1\right)}^{th}$ and the *s*^th^ pose feature segments.

#### 3.3.1. Offline Model Training

##### (1) Key Pose Extraction and Transition Probability Calculation

Key poses can be extracted from pose feature segments by use of clustering methods. The Gaussian Mixture Model (GMM) method is utilized to cluster key poses from pose feature segments in this study. The cluster number of key poses for each kind of human action is *K_p_*. 

After key poses $\left\{KP_{c}^{k}|k=1,...,K_{p}\right\}$ are obtained for human action *c*, the transition probabilities among key poses are needed to compute. Pose feature segments of training data of each human action are labeled with key poses by use of the nearest neighbor classifier. Given the obtained label sequences $\left\{L(s)\in [1,...,K_{p}]|s=1,2,...\right\}$, the transition probability between each two key poses can be calculated according to statistical results. 

##### (2) Atomic Motion Extraction

The extraction process of atomic motions is not independent with key poses, since atomic motions are considered as the transition process between two key poses. Thus, the extraction of atomic motions should be performed for each two key poses separately. The extraction of atomic motions can be described as follows:

Firstly, label each pose feature segment with the clustered key poses by use of the nearest neighbor classifier. The left half and right half of each pose feature segment are labeled as L*_lhalf_*(s) and *L_rhalf_*(*s*) respectively. It cannot be sure L*_lhalf_*(s) is equal to *L_rhalf_*(*s*) since *E_min_* is larger than zero. An example can be like “1-MS-2-2-MS-3-4-MS-4-3-MS-5” where each MS denotes one motion feature segment and the numbers denote key pose labels of pose feature segments. 

Secondly, classify motion feature segments which have the same “a-MS-b” pattern (*i.e.*, *L_rhalf_*(*s*–1) = *a* and *L_lhalf_*(*s*) = *b*) into same motion cluster *MC*(*a,b*), *a*∈(1,2,…,*K_p_*), *b*∈(1,2,…,*K_p_*). 

**Figure 4:**
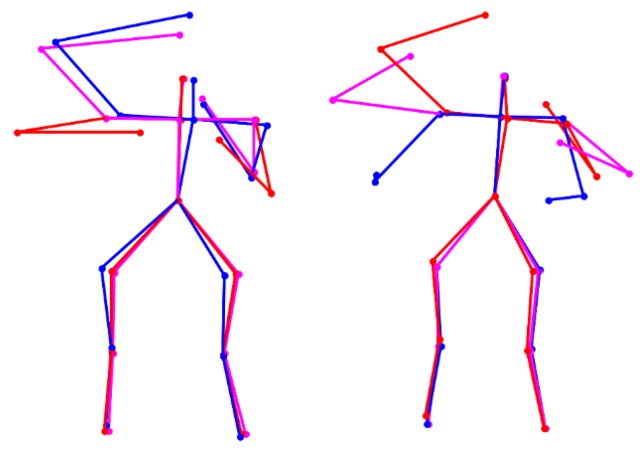
Visualization of two atomic motions of the action “wearing contact lenses” in CAD-60 dataset. The three time-ordered feature vectors are visualized as the red, magenta, and blue skeleton, respectively.

Lastly, extract atomic motions from each motion cluster *MC*(*a,b*). For each motion feature segment in one motion cluster, extract feature vectors located in the left-hillside/peak/right-hillside positions of the potential difference curve (e.g., positions *L*/*P*/*R* in Figure 3) respectively. Then, average the extracted feature vectors located in the three kinds of positions respectively, and thus three averaged feature vectors are obtained. At last, the atomic motion is represented by the three time-ordered feature vectors. A visualization example of atomic motions is displayed in Figure 4.

Up to now, the extraction methods of key poses and atomic motions have been elaborated, and the offline model training process for each human action is completed. Each human action is represented by key poses and atomic motions extracted from the “Feature Extraction-Segmentation-Clustering” training processes. 

#### 3.3.2. Online Model Matching

In the online CHAR, skeletal data are being generated frame by frame. The online model matching process is performed on the segments generated by the online segmentation method, so the operations described in this section will be executed before Step 3 in Algorithm 1 when one new segment is completed in Step 2. 

The online model matching process is actually likelihood probability calculation process, including likelihood probabilities that one pose feature segment is recognized to be the relevant key poses, and likelihood probabilities that one motion feature segment is recognized to be the relevant atomic motions. Given the assumption that there may be *C* kinds of human actions at most in one to-be-recognized data sequence, and each human action has *K_p_* key poses extracted in the training stage, the likelihood probabilities of the *s*^th^ pose feature segment based on the *C* × *K_p_* key poses can be calculated as:

(5) $$PDist_{s}(c,k)=\sum_{i=1}^{N_{s}}{\left\|F_{s}^{i}-KP_{c}^{k}\right\|}^{2}$$

(6) $$P_{kp}^{s}(c,k)=\frac{\frac{1}{PDist_{s}(c,k)}}{\sum_{c=1}^{C}\sum_{k=1}^{K_{p}}\frac{1}{PDist_{s}(c,k)}}$$

where $F_{s}^{i}$ is the *i*^th^ feature vector in the *s*^th^ pose feature segment, *N_s_* is the total count of feature vectors within the segment, and *KP_c_^K ^* is the *k*^th^ key pose of the *c*^th^ human action. The *PDist_s_*(*c,k*) is the distance between the *s*^th^ segment and the *k*^th^ key pose of the *c*^th^ human action, and the $P_{kp}^{s}(c,k)$ is the likelihood probability that the *s*^th^ segment is recognized as the *k*^th^ key pose of the *c*^th^ human action.

Similarly, the likelihood probabilities of one motion feature segment can be calculated as:

(7) $$MDist_{s}(c,k_{1},k_{2})=\frac{N_{s}}{N_{mv}}{\left\|MV_{s}-AM_{c}^{<k_{1},k_{2}>}\right\|}^{2}$$

(8) $$P_{am}^{s}(c,k_{1},k_{2})=\frac{\frac{1}{MDist_{s}(c,k_{1},k_{2})}}{\sum_{c=1}^{C}\sum_{k_{1}=1}^{K_{p}}\sum_{k_{2}=1}^{K_{p}}\frac{1}{MDist_{s}(c,k_{1},k_{2})}}$$

The $MV_{s}$ is the motion vector composed of three ordered feature vectors of the *s*^th^ motion feature segment, whose frame indices are indicated by the symbols L, P, R, as illustrated in Figure 3. The $AM_{c}^{<k_{1},k_{2}>}$ is the atomic motion associated with the $k_{1}^{th}$ and the $k_{2}^{th}$ key poses of the $c^{th}$ human action. $N_{s}$ is the total count of the feature vectors within the segment, and $N_{mv}=3$ is the frame number of $MV_{s}$. The $MDist_{s}(c,k_{1},k_{2})$ is the distance, and the $P_{am}^{s}(c,k_{1},k_{2})$ is the likelihood probability that the *s*^th^ segment is recognized as the atomic motion associated with the $k_{1}^{th}$ and $k_{2}^{th}$ key poses of the $c^{th}$ human action.

In summary, the probabilities $\left\{P_{kp}^{s}(c,k)|c=1,...,C;k=1,...,K_{p}\right\}$ are computed if the new segment is one pose feature segment, and the probabilities $\left\{P_{am}^{s}(c,k_{1},k_{2})|c=1,...,C;k_{1}=1,...,K_{p};k_{2}=1,...,K_{p}\right\}$ are computed if the new segment is one motion feature segment, in the online model matching process. The $P_{kp}^{s}(c,k)$ and $P_{am}^{s}(c,k_{1},k_{2})$ will be the basic elements in the utilized variable-length MEMM model.

### 3.4. Classification with Variable-Length MEMM

A human action can be intuitively modeled by a sequence of key poses and atomic motions in a particular order [16]. Key poses may be discriminatory or neutral, so are atomic motions. Sometimes humans could recognize one human action just because of one pose or one movement, but sometimes humans cannot tell the category of one human action until the last pose or movement happens. In such circumstances, the traditional Supported Vector Machine (SVM) [8] or Random Forest (RF) [41] methods which have the same feature dimensionality among training features and testing features may encounter their limitations. The Dynamic Time Warping (DTW) [42] method and the Hidden Markov Model (HMM) [43] method can take full use of the temporal sequence information among key poses and atomic motions, but they reduce the robustness to intra-class variety. Furthermore, they do not distinguish different roles of discriminatory and neutral key poses. 

**Figure 5:**
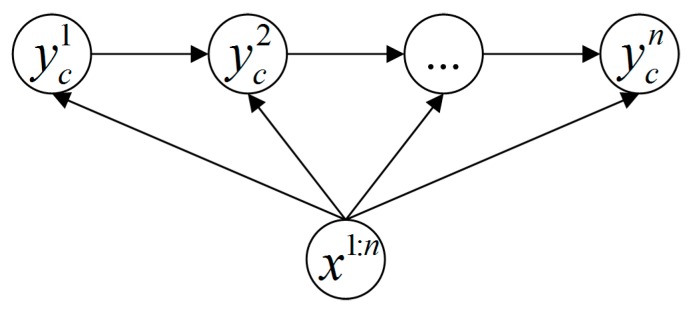
Maximum Entropy Markov Model.

In this study, we employ a variable-length MEMM method to recognize human actions dynamically based on the likelihood probabilities computed in the online model matching process. As illustrated in Figure 5, the traditional MEMM tries to find $P_{1:n}(c)=\max P(y_{c}^{1},y_{c}^{2},...,y_{c}^{n}|x^{1:n})$ where $y_{c}^{n}$ is one of the labels of the subclasses belonging to class *c*. However, our variable-length MEMM method does not calculate $P_{1:n}(c)$ with constant step count directly, it runs as in Algorithm 2.

**Algorithm 2.** The variable-length MEMM process**For**
$l=1,...,L_{\max}$ /* $L_{\max}$ is the largest step count */   **For**
c=1,2,...,C
  Calculate $P_{n-l+1:n}(c)=\max P(y_{c}^{n-l+1},...,y_{c}^{n}|x^{n-l+1:n})$;   **End**
  **If**
$\max P_{n-l+1:n}(c)$ is significantly larger than others    **return**
$\arg\underset{c}{\max}\;P_{n-l+1:n}(c)$; **End**
**End** **return**
$\arg\underset{c}{\max}\;P_{n-l+1:n}(c)$;

Let $O_{n,l}=\{x_{m}^{s},x_{p}^{s}|s=n-l+1,...,n\}$ denote the features within the feature segments indexed from n−l+1 to *n*, $x_{m}^{s}$ represent the feature vectors of the $s^{th}$ motion feature segment, and $x_{p}^{s}$ represent the feature vectors of the $s^{th}$ pose feature segment. Since one human action can be modeled as a sequence of key poses and atomic motions, the probability $P_{n-l+1:n}(c)$ in Algorithm 2 can be computed as

(9) $$P_{n-l+1:n}(c)=P(y_{c}^{n-l+1},...,y_{c}^{n}|O_{n,l})$$

where $y_{c}^{s}\in [KP_{c}^{1},KP_{c}^{2},...,KP_{c}^{K_{p}}],s=n-l+1,...,n$ is one key pose of the $c^{th}$ human action. Then, the joint probability $P(y_{c}^{n-l+1},...,y_{c}^{n}|O_{n,l})$ can be computed according to MEMM principle as

(10) $$\begin{array}{l} P(y_{c}^{n-l+1},...,y_{c}^{n}|O_{n,l})\\ \begin{array}{cc} & \begin{array}{l} =\prod_{s=n-l+2}^{n}P(y_{c}^{s}|y_{c}^{s-1},x_{m}^{s},x_{p}^{s})\\ \begin{array}{cc} & \cdot \sum_{y_{c}^{n-l}}P(y_{c}^{n-l+1}|y_{c}^{n-l},x_{m}^{n-l+1},x_{p}^{n-l+1})P(y_{c}^{n-l}) \end{array} \end{array} \end{array} \end{array}$$

According to Bayes rule, we get

(11) $$P(y_{c}^{s}|y_{c}^{s-1},x_{m}^{s},x_{p}^{s})=\frac{P(y_{c}^{s-1},x_{m}^{s},x_{p}^{s}|y_{c}^{s})P(y_{c}^{s})}{P(y_{c}^{s-1},x_{m}^{s},x_{p}^{s})}$$

By referring to [14], we also make a naive Bayes conditional independence assumption that $y_{c}^{s-1}$ is independent from $x_{m}^{s}$ and $x_{p}^{s}$ given $y_{c}^{s}$, then we get

(12) $$\begin{array}{l} P(y_{c}^{s}|y_{c}^{s-1},x_{m}^{s},x_{p}^{s})\\ \begin{array}{l} \begin{array}{cc} & =\frac{P(y_{c}^{s-1}|y_{c}^{s})P(x_{m}^{s},x_{p}^{s}|y_{c}^{s})P(y_{c}^{s})}{P(y_{c}^{s-1})P(x_{m}^{s},x_{p}^{s})} \end{array}\\ \begin{array}{cc} & =\frac{P(y_{c}^{s}|y_{c}^{s-1})P(y_{c}^{s}|x_{m}^{s},x_{p}^{s})}{P(y_{c}^{s})} \end{array} \end{array} \end{array}$$

The transition probability $P(y_{c}^{s}|y_{c}^{s-1})$ has been computed in the offline model training process. $P(y_{c}^{s}|x_{p}^{s},x_{m}^{s})$ can be computed as

(13) $$\begin{array}{cc} P(y_{c}^{s}|x_{m}^{s},x_{p}^{s})=P_{am}^{s}(c,k_{1},k_{2})P_{kp}^{s}(c,k_{2}) & where \end{array}\{\begin{array}{c} y_{c}^{s-1}=k_{1}\\ y_{c}^{s}=k_{2} \end{array}$$

The online segmentation method limits the maximal length of pose feature segments in order to reduce the delay of online human action recognition. As a result, there may have continuous pose feature segments. In such case, Equation (13) is degraded into

(14) $$\begin{array}{cc} P(y_{c}^{s}|x_{m}^{s},x_{p}^{s})=P(y_{c}^{s}|x_{p}^{s})=P_{kp}^{s}(c,k) & where \end{array}y_{c}^{s}=k$$

Up to now, the joint probability $P(y_{c}^{n-l+1},...,y_{c}^{n}|O_{n,l})$ can be calculated based on $P_{kp}^{s}(c,k)$, $P_{am}^{s}(c,k_{1},k_{2})$, and $P(y_{c}^{s}|y_{c}^{s-1})$. $P_{kp}^{s}(c,k)$ and $P_{am}^{s}(c,k_{1},k_{2})$ are calculated during the online model matching process, and $P(y_{c}^{s}|y_{c}^{s-1})$ is obtained in the offline model training process. Finally, human actions can be recognized by use of the variable-length MEMM according to Equations (9)–(14) and the outline illustrated in Algorithm 2. 

**Figure 6:**
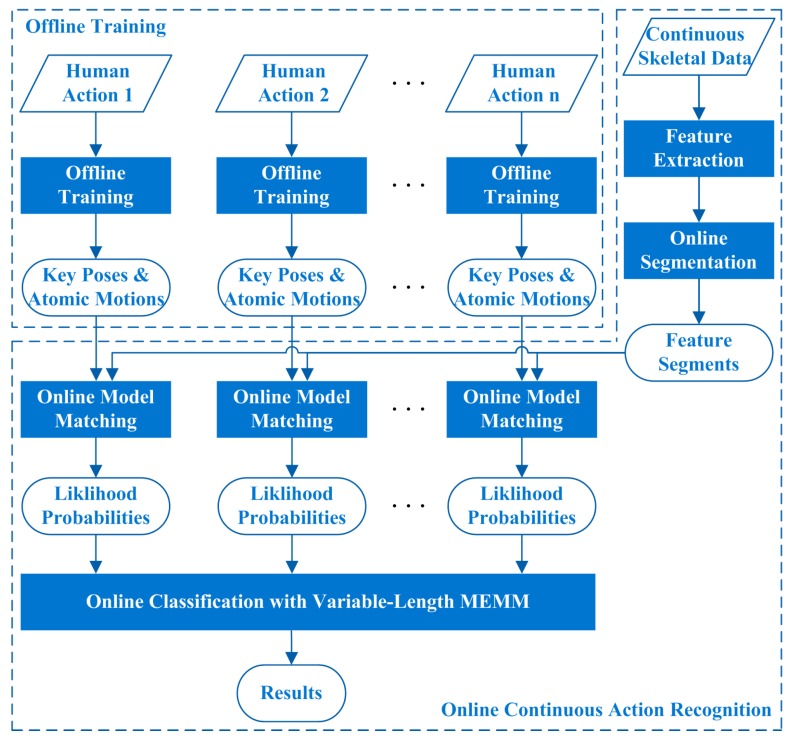
The outline of the proposed algorithm.

In summary, the outline of the proposed algorithm can be illustrated in Figure 6. The online continuous action recognition stage includes feature extraction, online segmentation, online model matching, and online classification with the variable-length MEMM, as shown in Figure 1 and Figure 6. In the online continuous action recognition stage, the online model matching and online classification with variable-length MEMM will be executed before Step 3 in Algorithm 1, when at least one new segment is completed during Step 2 in Algorithm 1. The offline training stage includes feature extraction, segmentation, and offline model training, as shown in Figure 1. In the offline training stage, key poses and atomic motions of each human action can be extracted in the offline model training process, after the segmentation process is done completely.

## 4. Experimental Results and Discussion

### 4.1. Experimental Setup

In our evaluation, the Cornell CAD-60 dataset and MSR Daily Activity 3D dataset are utilized to demonstrate the effectiveness of the proposed algorithm. These datasets are captured by a Kinect sensor, and only skeletal data extracted from RGB-D images are utilized in our experiments. The CAD-60 dataset focuses on daily activities, including twelve kinds of daily activities in five different locations performed by four persons. The twelve kinds of daily activities can be listed in alphabetical order as: *“brushing teeth”*, *“cooking (chopping)”*, *“cooking (stirring)”*, *“drinking water”*, *“opening pill container”*, *“relaxing on couch”*, *“rinsing mouth with water”*, *“talking on couch”*, *“talking on the phone”*, *“wearing contact lenses”*, *“working on computer”*, *“writing on whiteboard”*. The frame rate is 30 fps, and the length of each data sequence is about 45 s. The total number of activity sequences in CAD-60 is 60. The MSR Daily Activity 3D dataset focuses on sixteen kinds of activities performed by ten persons in an indoor environment: “*drink*”, “*eat”*, “*read book*”, “*call cell phone*”, “*write on a paper*”, “*use laptop*”, “*use vacuum cleaner*”, “*cheer up*”, “*sit still*”, “*toss paper*”, “*play game*”, “*lie down on sofa*”, “*walk*”, “*play guitar*”, “*stand up*”, *and* “*sit down*”. Each person performs each activity twice: one in sitting pose, and the other in standing pose. The total number of activity sequences in MSR Daily Activity 3D is 320. Since each data sequence in these datasets only includes one human action performed by one single person, we combine test data sequences into one continuous data sequence which contains multiple kinds of human actions. We perform the proposed algorithm on the continuous data sequence without extra supplementary marks in the online continuous action recognition evaluation stage. 

Frame-level accuracy is utilized as the criterion to evaluate the proposed algorithm and the published methods. Each continuous data sequence is labeled frame by frame beforehand, and the online continuous recognition result is also in frame-level. Thus, the frame-level accuracy is calculated as the ratio between the count of correctly classified frames and the total frame count of data sequences. 

The count of key poses for each human action is *K_p_* = 9, the segmentation threshold *E_min_* is set to 0.015, the maximal length of pose feature segments is *LP_max_* = 60 (*i.e.*, 2 s), and the minimal length *LM_min_* of motion feature segments is set to five frames. The first frame of feature vectors in each continuous data sequence is chosen as the referred feature vector for the sequence. The experiments are executed on the computer which has an Intel Core i5-4570 3.20 GHz processor and Windows 7 operating system. MATLAB 2012b is employed as the simulation platform, and the used GMM function is built-in function of MATALB. The MATLAB mixed programming with C language is utilized to improve the efficiency of the proposed algorithm.

### 4.2. Segmentation Evaluation on CAD-60

Different from the published CHAR methods [18,19,20], the proposed algorithm does not detect the start and end points of each human action. We divide feature sequences into pose feature segments and motion feature segments, and thus poses and movements of each human action are embedded in the feature segments. The proposed algorithm tries to label each feature segment with correct human action category by use of the variable-length MEMM, thus the segmentation results do matter to the action recognition result. 

**Figure 7:**
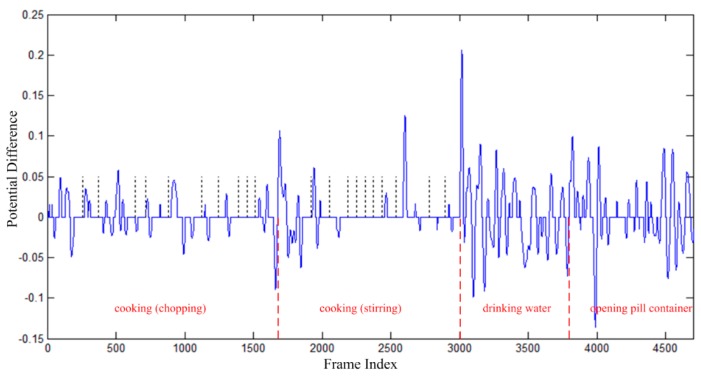
Segmentation results of one continuous data sequence. (The blue curves denote motion feature segments, the blue horizontal lines between each two blue curve parts indicate the pose feature segments, the black vertical dotted lines indicate the boundaries of pose feature segments, the red vertical dashed lines indicate the boundaries of different kinds of human actions.)

A segmentation example is illustrated in Figure 7. The continuous data sequence includes four kinds of human activities, *i.e.*, “*cooking (chopping)*”, “*cooking (stirring)*”, “*drinking water*”, and “*opening pill container*”. Each blue curve indicates one motion feature segment, and each blue horizontal line between blue curves indicate one pose feature segment. As aforementioned in Algorithm 1, long pose feature segments will be divided into short pose feature segments, and the boundaries are indicated by black vertical dotted lines in Figure 7. The boundaries between different kinds of human actions are indicated by red vertical dashed lines. It can be found from Figure 7 that the boundaries of human actions are located on the boundaries of feature segments in some degree. This means that human actions can be distinguished in feature sequences, and correct classification of feature segments will result in correct recognition of human actions. 

It is particularly necessary to point out that it does not mean that there must be no movements in the time intervals of pose feature segments, tiny movements may happen, e.g., the tiny movements when “cooking (stirring)”. However, tiny movements may be noises, and they carry less information for action recognition. Although sometimes tiny movements may also carry important information for some human actions, the unstableness of skeletal data under the condition of limited sensor accuracy makes them indistinguishable in some degree. Therefore, the proposed algorithm only focuses on significant movements.

### 4.3. Recognition Evaluation on CAD-60

Leave-one-person-out cross validation is utilized for the recognition evaluation on the CAD-60 dataset. We combine data sequences of the CAD-60 dataset performed by the same one person into one continuous data sequence, thus each continuous data sequence contains multiple kinds of human actions. We evaluate the proposed algorithm by recognizing human actions from the continuous data sequences, respectively. The misrecognition mainly occurs in two scenarios: The first is when human actions change suddenly, the first one or two feature segments of new human action may be misclassified if they are not distinguishable enough. Because the information of the previous segments which belong to the previous human action will be utilized together to recognize the one or two feature segments. The second is when some key poses or atomic motions of the misrecognized human actions are indistinguishable in some degree. For example, in human actions “drinking water” and “talking on the phone”, it is indistinguishable between key pose “keeping the cup around the mouth” and “keeping the phone around the ear” just from skeletal data only. 

**Figure 8:**
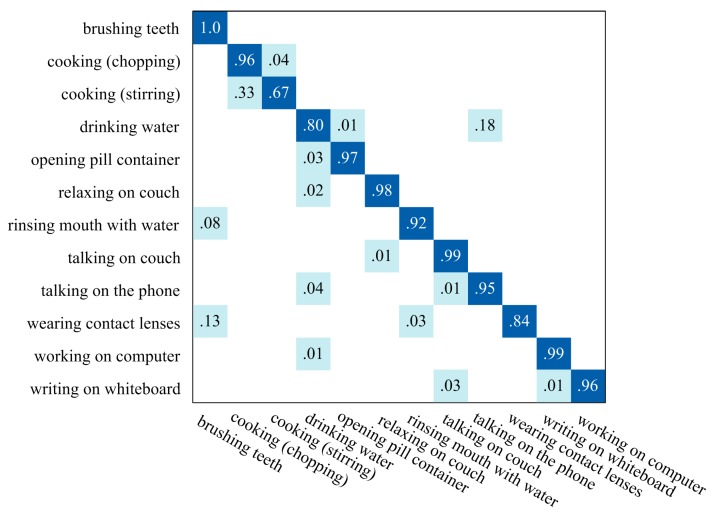
The average confusion matrix for recognition results of all data sequences in CAD-60.

Figure 8 illustrates the average confusion matrix for recognition results of all the continuous data sequences. All the recognition ratios have been rounded, so the ratio value “1.0” only means the misrecognition ratio of human action is less than 0.5%. The average accuracy of recognition results of all the continuous data sequences is 92.0%, which makes the proposed algorithm be an excellent online CHAR method. Since no published works have evaluated their CHAR methods on the CAD-60 dataset to the best of our knowledge, the proposed algorithm still obtain remarkable performances compared with the segmented human action recognition methods [41,44,45] which are performed on segmented data sequences, as illustrated in Table 1.

**Table 1:** Comparison of continuous human action recognition results on CAD-60 dataset.

Method	Accuracy
Our algorithm	**92.0%**
Pose Kinetic Energy [41]	91.9%
Actionlet [44]	74.7%
Sparse Coding [45]	65.3%

The misrecognition rate of “*cooking (stirring)*” is relative high, as displayed in Figure 8. This is because some key poses and movements in human actions “*cooking (chopping)*” and “*cook (stirring)*” are not very distinguishable without taking human-object interaction features into consideration. The same problem appears between human actions “*drinking water*” and “*talking on the phone*”.

The features which belong to different human actions can be divided into different segments, as illustrated in Figure 7. Thus, the recognition of feature segments does matter to the final action recognition result. We evaluated the recognition result of the first feature segment within each individual human action. “Correct Recognition” means that the first feature segment of the new human action is correctly-recognized instantly when the segment is obtained. “Delayed Recognition” means that the first one or two feature segments of the new human action are wrongly-recognized as the previous human action category, but the follow-up several segments are correctly-recognized. “Error Recognition” means that the first several feature segments within the new human action are all wrongly-recognized. We count up one when each of the three cases happens once, and the result is illustrated in Table 2. The count of “Delayed Recognition” is high, this is because some human actions cannot be recognized just according to one key pose or atomic motion, since one key pose or atomic motion does not contain enough context information, and some human actions are not so distinguishable without enough context information. 

**Table 2:** The recognition result of the boundaries between continuous actions.

Result	Correct Recognition	Delayed Recognition	Error Recognition
Count	27	36	2

### 4.4. Recognition Evaluation on MSR Daily Activity 3D

Only skeletal data is utilized when evaluating the proposed algorithm on the MSR Daily Activity 3D dataset. Each action is performed by 10 subjects for twice, the data from half of the subjects are used for training, and the other half for testing. Frame-level accuracy is utilized to evaluate the performance of the proposed algorithm and the published algorithms. The comparison results are illustrated in Table 3. 

**Table 3:** Comparison of continuous human action recognition results on MSR Daily Activity 3D dataset.

Method	Accuracy
Our algorithm	54.7%
Discriminative Orderlet [37]	60.1% ^a^
DSTIP + DCSF [46]	24.6%
EigenJoints [47]	47.0%
Moving Pose [30]	45.2%

^a^ Human-object interaction features are used in [37], but not in the proposed algorithm.

It can be found from Table 3 that the algorithm in [37] outperforms the proposed algorithm. This is because the algorithm in [37] is designed for recognition of human-object interaction and it utilizes human-object interaction features besides skeletal data of human body, but the proposed algorithm only utilizes skeletal data. All the sixteen kinds of activities in the MSR Daily Activity 3D dataset happen in the same indoor environment, and some of the activities are very similar without considering human-object interaction features. For example, the activities “drink”, “eat”, “call cell phone” have some very similar key poses, and it is even difficult for humans to distinguish them just according to skeletal data only. 

Compared with the recognition result on the CAD-60 dataset, the recognition accuracy on the MSR Daily Activity 3D dataset is relative low. This can be explained from the experimental setting and the dataset property. Firstly, leave-one-person-out cross-validation is used for CAD-60, but half of subjects are used for training and the other half for testing when evaluating on MSR Daily Activity 3D. Secondly, activities in five different locations in CAD-60 are recognized respectively, but all sixteen activities in MSR Daily Activity 3D are recognized in the same experiment and some of them are a little indistinguishable only with skeletal data of human body. Thirdly, the skeletal data of activities in CAD-60 are more distinguishable than those in MSR Daily Activity 3D when human-object interaction features are not taken into consideration. 

### 4.5. Efficiency Evaluation

In order to improve the efficiency of the proposed algorithm, the mixed programming between MATLAB and C language is utilized in the simulation experiments. The average processing time for each frame in the proposed algorithm is about 0.1 ms. Of course, the time for extraction of skeletal data from RGB-D images has not been counted in, since skeleton extraction is not in the scope of this study. Even so, the proposed algorithm can still be considered as an effective and efficient online CHAR method. 

### 4.6. Discussion

The evaluation results on CAD-60 show that the proposed algorithm can recognize new human actions instantly in most cases when one or two feature segments of new human actions are obtained. This demonstrates that the proposed variable-length MEMM method can take full use of different roles of discriminatory and neutral key poses or atomic motions in human action recognition, and can utilize temporal sequence information among key poses and atomic motions more effectively. It not only can ensure the recognition effectiveness of similar human actions by use of long MEMM, but also can improve the recognition efficiency of significant discriminatory human actions by use of short MEMM.

The evaluation results on the MSR Daily Activity 3D dataset demonstrate that sometimes it is not enough to distinguish human actions which have similar key poses just based on skeletal data only, and human-object interaction features can be utilized to improve the effectiveness of human action recognition further.

The proposed algorithm represents a human action by a sequence of key poses and atomic motions in a particular order. It is consistent with human perception models on human actions and the way that humans describe one human action with natural language. Besides, it is more reasonable to divide feature sequences into pose feature segments and motion feature segments to extract key poses and atomic motions more precisely, compared with the published methods extracting key poses from feature sequences directly. 

## 5. Conclusions

In this paper, an online continuous human action recognition algorithm is proposed. The proposed algorithm does not need to detect the start and end points of each human action in advance. The proposed algorithm only uses skeletal data extracted from RGB-D images, and performs online model matching process and online action recognition process on feature segments obtained by online segmentation method. These two factors make the proposed algorithm high efficient. Besides, the variable-length maximal entropy Markov model method ensures the effectiveness and efficiency on continuous human action recognition. 

However, the proposed algorithm still extracts features of the whole human body, and trains the models based on whole body features. Maybe it will be more reasonable to extract features for each limb separately and train hierarchical models based on human limb structures. Besides, skeletal data has limitations for universal and practical human action recognition, thus it will be more reasonable to recognize human actions by integrated utilization of RGB-D images and skeletal data, and human-object interaction features should also be taken into consideration. These will be our future work.
